# ROMP-Derived cyclooctene-based monolithic polymeric materials reinforced with inorganic nanoparticles for applications in tissue engineering

**DOI:** 10.3762/bjoc.6.137

**Published:** 2010-12-17

**Authors:** Franziska Weichelt, Solvig Lenz, Stefanie Tiede, Ingrid Reinhardt, Bernhard Frerich, Michael R Buchmeiser

**Affiliations:** 1 Leibniz-Institut für Oberflächenmodifizierung e. V. (IOM), Permoserstrasse 15, D-04318 Leipzig, Germany; 2 Klinik und Poliklinik für Mund-, Kiefer- und Plastische Gesichtschirurgie, Universität Rostock, Schillingallee 35, D-18057 Rostock, Germany; 3 Institut für Polymerchemie, Lehrstuhl für Makromolekulare Stoffe und Faserchemie, Universität Stuttgart, Pfaffenwaldring 55, D-70569 Stuttgart, Germany; 4 Institut für Textilchemie und Chemiefasern, Körschtalstrasse 26, D-73770 Denkendorf, Germany

**Keywords:** hybrid materials, monoliths, nanoparticles, ring-opening metathesis polymerization (ROMP), tissue engineering

## Abstract

Porous monolithic inorganic/polymeric hybrid materials have been prepared via ring-opening metathesis copolymerization starting from a highly polar monomer, i.e., *cis*-5-cyclooctene-*trans-*1,2-diol and a 7-oxanorborn-2-ene-derived cross-linker in the presence of porogenic solvents and two types of inorganic nanoparticles (i.e., CaCO_3_ and calcium hydroxyapatite, respectively) using the third-generation Grubbs initiator RuCl_2_(Py)_2_(IMesH_2_)(CHPh). The physico-chemical properties of the monolithic materials, such as pore size distribution and microhardness were studied with regard to the nanoparticle type and content. Moreover, the reinforced monoliths were tested for the possible use as scaffold materials in tissue engineering, by carrying out cell cultivation experiments with human adipose tissue-derived stromal cells.

## Introduction

Tissue engineering (TE), a sub-area of regenerative medicine, brings together diverse technologies and interdisciplinary fields such as biology, engineering, material and life sciences, polymer and inorganic chemistry [1–3]. Its general task is the development of functional substitutes for the replacement or restoration of tissue or organ function with scaffolds containing specific populations of living cells [4–5]. After the cultivation of cells on such biological substitutes, they are subsequently applied to living organisms, where they ideally should restore, maintain or improve tissue function or whole organs [2–3]. A challenging task in this context is the development of suitable scaffold materials, which can act as matrices for the delivery of the cells to defect sites, with desired properties such as adequate pore size and pore structure, biocompatibility, biodegradability or mechanical strength.

Due to their unitary porous structure and the ease of synthesis via polymerization or consolidation processes, monolithic materials were introduced into the field of TE some years ago [6–7]. Several studies have shown that the properties of the scaffold material, such as mechanical properties, porosity or surface structure, strongly affect the differentiation of mesenchymal stem cells (i.e., the formation of osseous, muscle or neural cells) and especially for the differentiation into osteoblasts, stiff materials are required [7–12]. Bone is a natural composite material, being composed of an inorganic compound (calcium hydroxyapatite) incorporated into an organic matrix (collagen) and thus resulting in a material, which possesses high stiffness and fracture toughness [13–14].

Ring-opening metathesis polymerization (ROMP) derived norborn-2-ene (NBE)-based monolithic materials have previously been successfully tested for both osseous and adipose cell growth [6]. However, it has also been reported that the mechanical properties, such as hardness, of such scaffold materials were quite low. Harder materials with a specific surface structure and porosity, however, would allow for the differentiation of mesenchymal stem cells into osteoblasts, and thus the development of scaffold materials for bone regeneration. Ideal biomaterials for bone TE should be non-immunogeneic, biodegradable, highly osteoinductive and provide mechanical support when needed [15]. As an alternative to poly(NBE)-based monoliths, we investigated the preparation of monolithic structures from a highly polar cyclooctene derivative. So far, ROMP-derived monolithic materials have successfully been applied to separation science as well as heterogeneous catalysis [16–18]. Generally, cyclooctene-derived monoliths differ from their NBE-based counterparts in that they are less prone to oxidation and display higher elastic moduli. With the aim to synthesize organic-inorganic monolithic hybrid materials for application as scaffold materials in TE with specific properties, e.g., high mechanical strength and biocompatibility, we report here ROMP-derived cyclooctene-based monoliths reinforced with two of the most frequently used inorganic materials in nature, i.e., calcite and calcium hydroxyapatite [19], respectively.

## Results and Discussion

### Synthesis of CaCO_3_ and HAp

**Figure 1:**
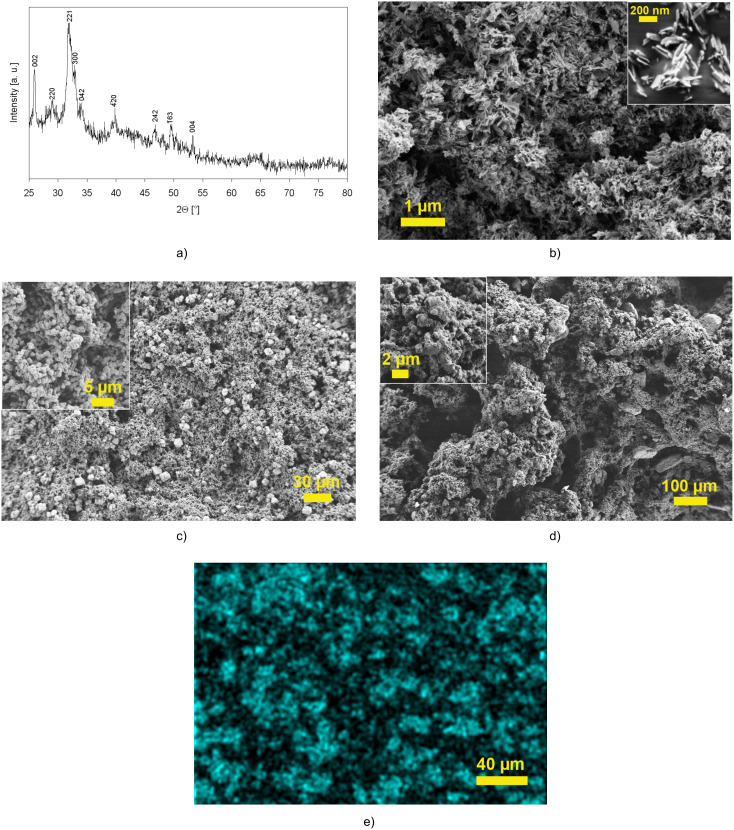
Nanoscale **HAp**: calcium phosphate hydroxide (Ca_5_(PO_4_)_3_OH) as evidenced by XRD measurements (a), SEM picture of the nanoparticles (b), structures of **COE**-derived monoliths containing 12 wt % **CaCO_3_** (c) and 12 wt % **HAp** (d), and EDX-measurements of a monolith containing 12 wt % **CaCO_3_** (Ca-mapping) (e).

Nanosized calcium carbonate (**CaCO_3_**) and calcium hydroxyapatite (**HAp**) were prepared by precipitation reactions in aqueous solution from CaCl_2_·2H_2_O and anhydrous Na_2_CO_3_, and from CaCl_2_·H_2_O, H_3_PO_4_ and NH_4_OH, respectively. For **CaCO_3_**, scanning electron microscopic (SEM) images of the powders showed agglomerates of nanoparticles (~50–100 nm) and a rhombohedral calcite crystal structure was detected both by X-ray diffraction (XRD) and Raman measurements. Nano-sized platelets with a thickness <20 nm were formed in the synthesis of **HAp** and exhibited a calcium phosphate hydroxide crystal structure again from XRD and Raman measurements (Figure 1).

### Preparation and characterization of monolithic materials

**Scheme 1:**
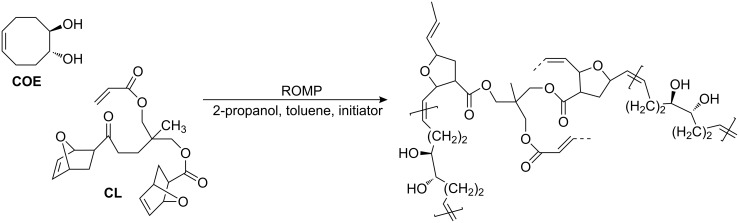
ROMP-based synthesis of *cis*-5-cyclooctene-*trans-*1,2-diol based polymeric monolithic scaffolds.

Monolithic hybrid materials were then prepared via ROMP from *cis*-5-cyclooctene-*trans-*1,2-diol (**COE**), a 7-oxanorborn-2-ene-derived cross-linker (**CL**) and up to 12 wt % of the inorganic compounds in the presence of a microporogen (toluene) and a macroporogen (2-propanol) under phase separation conditions using the third-generation Grubbs initiator RuCl_2_(Py)_2_(IMesH_2_)(CHPh) (IMesH_2_ = 1,3-dimesitylimidazolin-2-ylidene, Py = pyridine) (Scheme 1, Table 1).

**Table 1:** Compositions of *cis*-5-cyclooctene-*trans-*1,2-diol (**COE**)-based monoliths.

CL^a^	COE^a^	Toluene^a^	2-Propanol^a^	Nanoparticles^a^

22.5	22.5	9	46	—
26	26	9	33	6 (CaCO_3_)
24	24	9	31	12 (CaCO_3_)
26	26	9	33	6 (HAp)
24	24	9	31	12 (HAp)

^a^in [wt %]; 0.07 wt % of initiator were used throughout.

*cis*-5-Cyclooctene-*trans-*1,2-diol was chosen as a highly polar monomer, which should together with the 7-oxanorborn-2-ene-based cross-linker form a polar polymeric matrix that facilitates the incorporation and homogeneous distribution of the polar nanoparticles. The cross-linker itself has already been reported to be biocompatible [7]. SEM images of the nanoparticle-reinforced monolithic materials revealed an increase of the pore sizes with increasing nanoparticle content. An increase of the pore size from 10–30 µm (for monoliths without any inorganic component) up to 25–70 µm was observed with the addition of 12 wt % **CaCO_3_**, while pores up to 130–450 µm were formed using 12 wt % nano-sized **HAp** (Figure 1). Thus, as reported previously [20], the inorganic nanoparticles serve as macroporogens in the phase-separation-triggered synthesis of the monolithic matrix. The adjustment of the pore size by variation of the nanoparticle content and type is advantageous and may allow for an alleviated cell attachment as well as ingrowth of living cells on the scaffold materials. Due to the OH groups present at its surface, **HAp** is more polar than **CaCO_3_** and thus affects the pore formation process more strongly, resulting in even larger pore size. Energy dispersive X-ray mapping (EDX) revealed a homogeneous and non-aggregated distribution of **CaCO_3_** in the polymeric matrix (Figure 1). In contrast, agglomerates of **HAp** up to 100 µm in size were found for **HAp**-based monoliths and also the distribution of the inorganic compound was less homogeneous, supporting the explanation of polar interactions between organic components and **HAp** in the polymerization mixture. Nitrogen adsorption measurements of the monolithic powders confirmed their highly porous structure, showing specific surface areas of between 2 m^2^/g (unfilled and **CaCO_3_**-reinforced monoliths) and 43 m^2^/g (**HAp**-reinforced monoliths). The microhardness of the monolithic materials was determined with a Vickers hardness measurement device. In comparison to the microhardness of unfilled **COE**-based monoliths (5.8 N/mm^2^), a more than 2-fold increase of the microhardness was observed with the addition of 6 wt % **CaCO_3_** (16.3 N/mm^2^) and **HAp** (12.4 N/mm^2^), which decreased with the addition of 12 wt % **CaCO_3_** (4.5 N/mm^2^) and **HAp** (8.1 N/mm^2^), respectively. The decrease of the microhardness with increasing nanoparticle content is attributed to the increase of pore size and porosity as explained above. Thus, with the addition of inorganic components, the microhardness of **COE**-based monoliths could efficiently be increased, however, the content of nanoparticles must not be too high, otherwise the effect of the larger pore size and porosity becomes dominant and the microhardness decreases. A decrease of the compressibility, i.e., an increase in the compressive force-compression (CF-C) ratio of 56% was observed for reinforcement with the addition of 12 wt % **CaCO_3_** (1.08 MPa for reinforced monolith vs 0.62 MPa for unmodified monolith). Thus, **CaCO_3_**-reinforced monoliths were more resistant to the pressure applied. In contrast, a strong reduction of the CF-C ratio (0.03 MPa) was observed on reinforcement with 12 wt % **HAp**, verifying a high compressibility of the **HAp**-based monoliths at simultaneously high microhardness.

### Cell cultivation experiments

**Figure 2:**
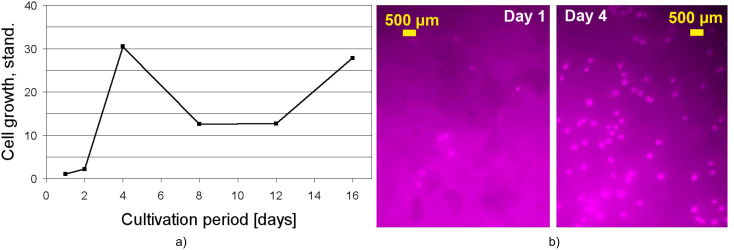
(a) Cell growth of human adipose tissue-derived stromal cells. Each data point is the average of 12 data points. (b) Fluorescence microscopy images of living cells after one and four days of cultivation; cells (human adipose tissue-derived stem cells) grown on a **COE**-based scaffold reinforced with 12 wt % **CaCO_3_**.

After careful washing and drying, the monolithic materials were cut into discs ~1–2 mm in height and ~10 mm in diameter and subsequently sterilized via γ-irradiation. Thereafter, these monolith scaffolds were seeded with human adipose tissue-derived stromal cells (hATSCs). Proliferation of the cells was monitored up to the 16^th^ day (Figure 2).

After placing the sterilized discs into 24-well plates, the culture medium was added and the samples were seeded with hATSCs with a density of 20,000 cells/cm^2^. Since only a part of the cells settled on the monolithic material, with the other part remaining in the surrounding culture medium, the cell number counted after the first day of cultivation was set as starting value for the proliferation curve. The determined cell numbers of the following days of cultivation were then divided by the starting value, in order to be able to compare the cell growth on different monolithic compositions. As it can be seen, a continuous increase of the cell number was observed up to the fourth day of proliferation, resulting in an increase of the cell number by a factor of 30 (709 cells/cm^2^ after day 1 vs 21,615 cells/cm^2^ after day 4). After the fourth day of proliferation the seeded scaffolds were transferred into 6-well plates, which probably affected the cell growth, and explains the decrease of the cell number between the fourth and eighth day of cultivation. Thereafter, proliferation continued with an increase of the cell number after the eighth day of cultivation. These data indicate a good biocompatibility of the monolithic hybrid support as well as sufficient cell adhesion on the monolithic material.

## Conclusion

**COE**-based monolithic scaffolds have been prepared via ROMP in the presence of two different types of inorganic nanoparticles. It was shown that variation of both the nanoparticle type and content affected the pore size of the monoliths, i.e., the pore size was the larger, the higher the content of the inorganic component. In addition, the mechanical properties of the monolithic structures could be modified with the addition of inorganic components (calcium carbonate and calcium hydroxyapatite, respectively). Preliminary cell cultivation experiments showed that the prepared monolithic hybrid materials can be cultivated with human adipose tissue-derived stromal cells. Current work focuses on the biodegradability of the novel scaffolds under physiological conditions.

## Experimental

IR spectra were recorded on a Bruker Vector 22 using ATR technology. Raman spectra were recorded on a Bruker RFS 100. Scanning electron microscopic (SEM) images were obtained on a Zeiss Ultra 55 field-emission (FEG) SEM device (Carl Zeiss SMT, Germany) at 0.6 keV for **CaCO_3_** and **HAp** powders and 1.0 keV for the monoliths. Powder X-ray diffraction (XRD) was performed on a Philips X’Pert wide-angle diffractometer with slit optics, Cu K_α_ radiation (λ = 154 nm) and Ni K_β_ filter. The powder was applied to the specimen holder using double faced-adhesive tape and the upper site was covered with a thin layer of the powder. Measurements were carried out at a voltage of 40 kV and a current of 30 mA in the 2Θ-range of 20–80° with a step size of 0.05 and 1.00 seconds per step. The microhardness was measured on a Fischerscope H 100 (Helmut Fischer GmbH + Co. KG, Germany). A load of 100 mN was applied on the sample for 20 s. For the determination of the Martens hardness, a Vickers diamond indenter was used. Ten measurements were made to get an average for the sample. A Sonics Vibra-Cell Ultrasonic Processor (Sonics & Materials, Inc., Newtown, USA) was used for sonication. The compressibility of the monoliths was tested at a Z 1120 zwicki (Zwick/Roell, Germany). The diameter of the specimens was dependent on monomer and nanoparticle content (~0.8–1.3 cm), the average height was 4 mm. The traverse was applied with a test rate of 1 mm/min. Due to problems in sample preparation, a perfect sample geometry was difficult to adjust. Hence, it could not be completely excluded that stresses during the compressibility measurements were exclusively induced by the applied pressure of the traverse, but could also arise due to inhomogeneities (porosity, microcracks) within the monoliths. Therefore, the compressibility here was defined as the compressive force-compression ratio (CF-C ratio) applied to the cross-sectional area.

CaCl_2_·2H_2_O (99%) and NH_4_OH (aqueous 26 wt % solution) were obtained from Riedel-de Haën. Anhydrous Na_2_CO_3_ (99%), anhydrous Na_2_SO_4_ (99%), NaHCO_3_ (99+%), ethyl vinyl ether (99%, stabilized), 1,1,1-tris(hydroxymethyl)ethane (97%) and 2-propanol (99.5%) were obtained from Acros Organics (Germany). NaOH (≥98%), CH_3_OH (99.8%) and H_3_PO_4_ (85%) were obtained from Fluka (Germany). CHCl_3_ (99.8%), dimethyl sulfoxide (DMSO, 99.9%), tetrahydrofuran (THF, 99.0%) and CH_2_Cl_2_ (≥99.8%) were obtained from Merck. Norborn-2-ene (99%) was obtained from Aldrich (Germany). Diethyl ether (99.9%), toluene (technical) and H_2_SO_4_ (95%) were obtained from BDH Prolabo (VWR, Germany). The cross-linker (**CL**) [6–7], *cis*-5-cyclooctene-*trans*-1,2-diol (**COE**) [21] and calcium carbonate (**CaCO_3_**) [22] were prepared according to literature procedures.

**HAp:** CaCl_2_^.^2H_2_O (13.26 g; 90.2 mmol) was dissolved in 900 mL of water. H_3_PO_4_ (85 wt % in water; 6.22 g; 53.9 mmol) was added and the solution heated to 90 °C. NH_4_OH (26 wt % in water) was added to give a pH of 9 and a white precipitate formed. The dispersion was cooled to room temperature, the precipitate collected by centrifugation and thoroughly washed with water. Finally, the powder was dried at 100 °C overnight. Yield: 6.41 g (71%). FT-IR (ATR mode) [20,23]: 3566 (w), 3221 (w), 1635 (w), 1402 (w, ν_(CO3)2-_), 1086 (m, ν_(PO4)3-_), 1014 (s, ν_(PO4)3-_), 960 (m, ν_(PO4)3-_), 866 (w) cm^−1^. Raman [24]: 429 (w, ν_(PO4)3-_), 587 (w, ν_(PO4)3-_), 961 (s, ν_(PO4)3-_), 1044 (w, ν_(PO4)3-_), 1073 (w, ν_(PO4)3-_) cm^−1^. XRD: Calcium phosphate hydroxide (Ca_5_(PO_4_)_3_OH, monoclinic; ref. card: 76-0694).

### Synthesis of monolithic scaffold materials [6–7]

Two solutions, A and B, were prepared and chilled to 0 °C. Solution A consisted of *cis*-5-cyclooctene-*trans-*1,2-diol, the CL, the macroporogen (2-propanol) and the inorganic filler (**CaCO_3_** and **HAp**, respectively; 0–12 wt %). Solution B was obtained by dissolving RuCl_2_(Py)_2_(IMesH_2_)(CHPh) (IMesH_2_ = 1,3-dimesitylimidazolin-2-ylidene, Py = pyridine; 0.07 wt %) in the microporogen (toluene, 9 wt %). Solutions A and B were mixed and stirred for a few seconds. The polymerization mixture was poured into 1 × 5 cm plastic devices. Polymer formation occurred within 12 hours in air. The monoliths were extensively washed with a mixture of CHCl_3_, DMSO and ethyl vinyl ether (2:40:28 wt %). Finally, they were washed with MeOH, dried in vacuo and cut into 1 × 1.5 cm pieces. A summary of the compositions is given in Table 1.

### Cultivation of adipose tissue-derived stromal cells

The harvesting and cultivation of human adipose tissue-derived stromal cells (hATSCs) has been previously described [25–26]. Briefly, small pieces of subcutaneous adipose tissue (<0.5 cm^3^) from the lateral thigh region were collected during elective surgery in the Department of Oral and Maxillofacial Surgery of the University of Rostock. The adipose tissue was minced with sterile scissors and subjected to collagenase digestion (collagenase type II, Boehringer, Mannheim, Germany). The suspension was centrifuged (300 g, 10 min) and plated in tissue culture flasks (Greiner, Frickenhausen, Germany). Cells were cultured in a 5% humidified CO_2_ atmosphere at 37 °C. Culture medium (“standard medium”: Iscove's MDM / HAM F12 1:1, supplemented with 10% neonatal calf serum (NCS), all from Life Technology, Paisley, Scotland) was changed every second day. The stem cell character of these cells has been previously demonstrated by successful osteogenic, adipogenic and smooth muscle differentiation [6–7,20,27]. The cells were split in a 1:4 ratio and amplified up to the third passage.

Sterilization of the samples was accomplished with γ-irradiation with the aid of a ^60^Co-γ-source at the Leibniz-Institute of Surface Modification (IOM, Leipzig, Germany) on a rotating table at a dose rate of 0.79 kGy/h. The dose rate was determined by Fricke-dosimetry. The reliability of the dose determination was checked against IAEA-alanine dosimeters resulting in an overall accuracy of the dose measurements of ±5%. The total dose applied was 24 kGy.

### Cell proliferation assay

Tests were made in 24-well-plates and 6-well-plates with a well diameter of 2 and 3.5 cm, respectively. Sterile discs of the monolithic test material (1–2 mm in height) were placed into the wells and covered with standard cell culture medium. After incubation in a humidified atmosphere at 37 °C for two days, the medium was removed and the discs were seeded. Cultures of third passage hATSC of three individuals were pooled for the experiment and seeded onto the discs at a density of 20,000 cells per cm². After 1, 2, 4, 8, 12 and 16 days of culture, the discs were harvested and fixed in 4 wt % formaldehyde in PBS (phophate buffered saline, Serva, Heidelberg, Germany) for 20 minutes. Discs were incubated in 4',6-diamidino-2-phenylindole (DAPI, Sigma-Aldrich, Steinheim, Germany) solution and examined under a fluorescent microscope (Axiovert 25, Zeiss, Jena, Germany). In every specimen, cells were counted in six fields and averaged.

## Supporting Information

File 1 IR and Raman spectra of HAp and CaCO_3_.

## References

[1] Puppi D, Chiellini F, Piras A M, Chiellini E (2010). Prog Polym Sci.

[2] Marler J J, Upton J, Langer R, Vacanti J P (1998). Adv Drug Delivery Rev.

[3] Nerem R M (1991). Ann Biomed Eng.

[4] Langer R, Vacanti J P (1993). Science.

[5] Cortesini R (2005). Transplant Immunol.

[6] Buchmeiser M R (2009). J Polym Sci, Part A: Polym Chem.

[7] Löber A, Verch A, Schlemmer B, Höfer S, Frerich B, Buchmeiser M R (2008). Angew Chem.

[8] Engler A J, Sen S, Sweeney H L, Discher D E (2006). Cell.

[9] Benoit D S W, Schwartz M P, Durney A R, Anseth A S (2008). Nat Mater.

[10] Lenhert S, Meier M-B, Meyer U, Chi L, Wiesmann H P (2005). Biomaterials.

[11] Liao H, Andersson A-S, Sutherland D, Petronis S, Kasemo B, Thomsen P (2003). Biomaterials.

[12] Gray C (1998). Tissue Eng.

[13] Weiner S, Wagner H D (1998). Annu Rev Mater Sci.

[14] Dorozhkin S V, Epple M (2002). Angew Chem, Int Ed.

[15] Du C, Cui F Z, Zhu X D, de Groot K (1999). J Biomed Mater Res.

[16] Bandari R, Knolle W, Buchmeiser M R (2008). J Chromatogr, A.

[17] Schlemmer B, Gatschelhofer C, Pieber T R, Sinner F M, Buchmeiser M R (2006). J Chromatogr, A.

[18] Buchmeiser M R (2002). Bioorg Med Chem Lett.

[19] Krenkova J, Lacher N A, Svec F (2010). Anal Chem.

[20] Weichelt F, Frerich B, Lenz S, Tiede S, Buchmeiser M R (2010). Macromol Rapid Commun.

[21] Bandari R, Prager-Duschke A, Kühnel C, Decker U, Schlemmer B, Buchmeiser M R (2006). Macromolecules.

[22] Wang A, Liu D, Yin H, Wu H, Wada Y, Ren M, Jiang T, Cheng X, Xu Y (2007). Mater Sci Eng, C.

[23] Okada M, Furuzono T J (2007). Nanopart Res.

[24] Silva C C, Sombra A S B (2004). J Phys Chem Solids.

[25] Frerich B, Kurtz-Hoffmann J, Lindemann N, Müller S (2000). Mund Kiefer GesichtsChir.

[26] Frerich B, Lindemann N, Kurtz-Hoffmann J, Oertel K (2001). Int J Oral Maxillofac Surg.

[27] Weinzierl K, Hemprich A, Frerich B J (2006). Cranio-Maxillofac Surg.

