# Supplementary material for: ROMP-Derived cyclooctene-based monolithic polymeric materials reinforced with inorganic nanoparticles for applications in tissue engineering
Source: Beilstein J Org Chem. 2010 Dec 17;6:1199–205. doi: 10.3762/bjoc.6.137 (PMC3028601; doi:10.3762/bjoc.6.137)
Supplement: File 1 — IR and Raman spectra of HAp and CaCO3. [file Beilstein_J_Org_Chem-06-1199-s001.pdf]

Supporting Information

for

ROMP-Derived cyclooctene-based monolithic polymeric materials reinforced with inorganic nanoparticles for applications in tissue engineering

Franziska Weichelt[1], Solvig Lenz[2], Stefanie Tiede[2], Ingrid Reinhardt[1], Bernhard Frerich[*2,§] and Michael R. Buchmeiser[*3,4,¶]

[1]Leibniz-Institut für Oberflächenmodifizierung e. V. IOM, Permoserstrasse 15, D-04318 Leipzig, Germany, [2]Klinik und Poliklinik für Mund-, Kiefer- und Plastische Gesichtschirurgie, Universität Rostock, Schillingallee 35, D-18057 Rostock, Germany, [3]Institut für Polymerchemie, Lehrstuhl für Makromolekulare Stoffe und Faserchemie, Universität Stuttgart, Pfaffenwaldring 55, D-70569 Stuttgart, Germany and [4]Institut für Textilchemie und Chemiefasern, Körschtalstrasse 26, D-73770 Denkendorf, Germany

Email : Bernhard Frerich - bernhard.frerich@med.uni-rostock.de; Michael R. Buchmeiser - michael.buchmeiser@ipoc.uni-stuttgart.de

* Corresponding author

§ Tel.: +49 (0) 381 494 6550; Fax: +49 (0) 381 494 6698

¶ Tel: +49 (0) 711 685 64075; Fax: +49 (0) 711 685 64050

IR and Raman spectra of HAp and $CaCO_3$

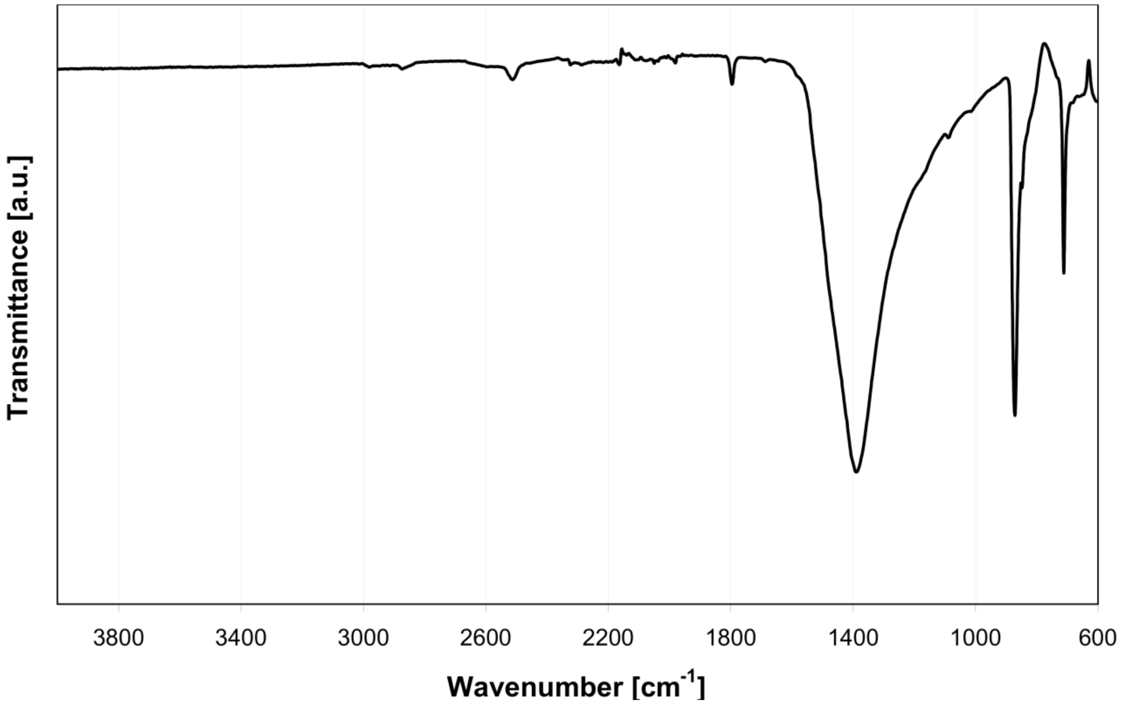

Figure S1: IR-spectum of **$CaCO_3$**.

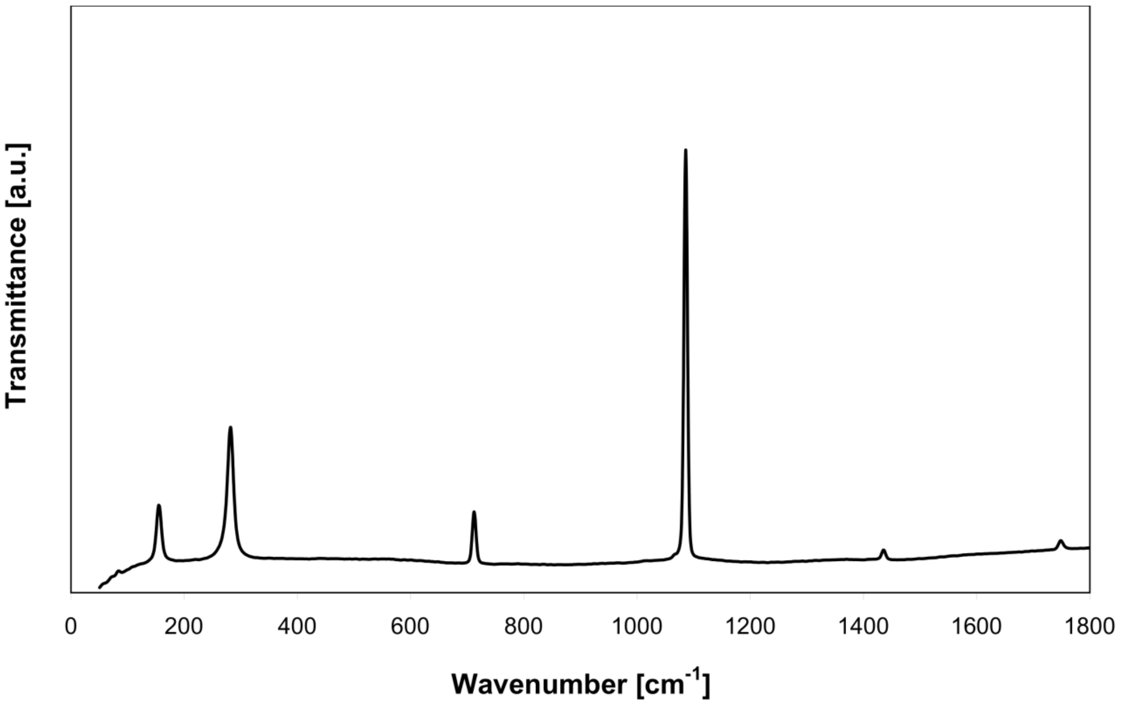

Figure S2: Raman spectrum of **$CaCO_3$**.

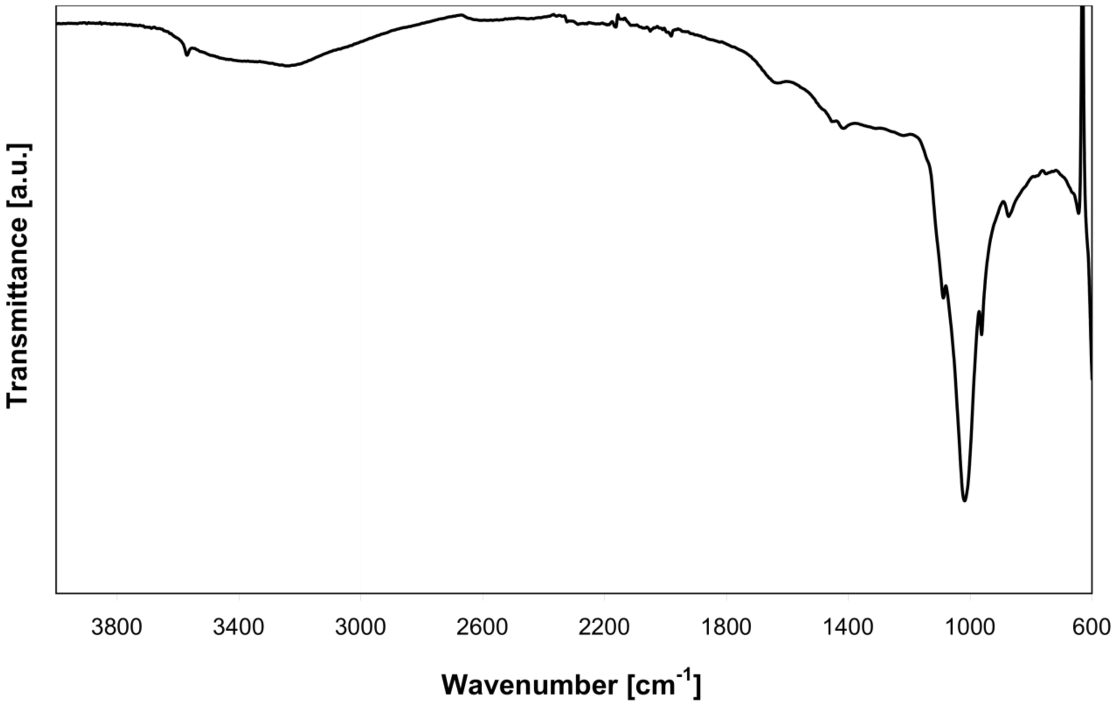

Figure S3: FT-IR spectrum of **HAp**.

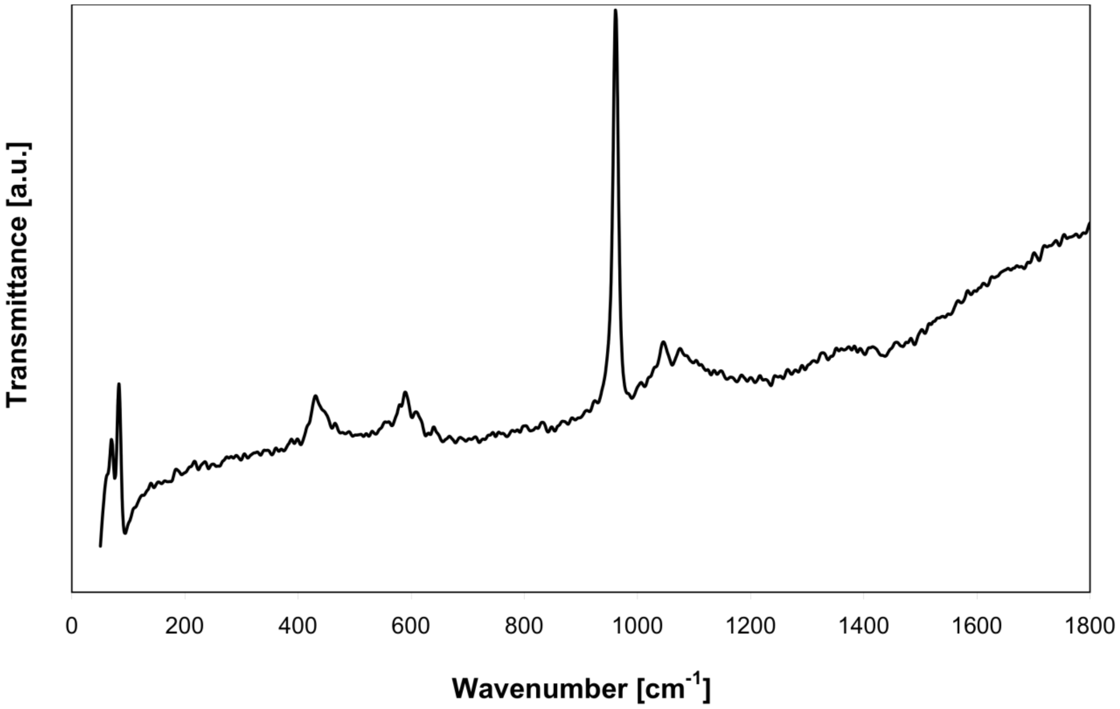

Figure S4: Raman-spectrum of **HAp**.
